# Not My Problem: Vicarious Conflict Adaptation with Human and Virtual Co-actors

**DOI:** 10.3389/fpsyg.2016.00606

**Published:** 2016-04-28

**Authors:** Michiel M. Spapé, Niklas Ravaja

**Affiliations:** ^1^Helsinki Institute for Information Technology HIIT, Aalto UniversityHelsinki, Finland; ^2^Helsinki Institute for Information Technology HIIT, University of HelsinkiHelsinki, Finland; ^3^Department of Social Research University of HelsinkiHelsinki, Finland; ^4^School of Business, Aalto UniversityHelsinki, Finland

**Keywords:** executive control, conflict adaptation, joint attention, Simon effect, task co-representation, social cognition, ECG

## Abstract

The Simon effect refers to an incompatibility between stimulus and response locations resulting in a conflict situation and, consequently, slower responses. Like other conflict effects, it is commonly reduced after repetitions, suggesting an executive control ability, which flexibly rewires cognitive processing and adapts to conflict. Interestingly, conflict is not necessarily individually defined: the Social Simon effect refers to a scenario where two people who share a task show a conflict effect where a single person does not. Recent studies showed these observations might converge into what could be called vicarious conflict adaptation, with evidence indicating that observing someone else's conflict may subsequently reduce one's own. While plausible, there is reason for doubt: both the social aspect of the Simon Effect, and the degree to which executive control accounts for the conflict adaptation effect, have become foci of debate in recent studies. Here, we present two experiments that were designed to test the social dimension of the effect by varying the social relationship between the actor and the co-actor. In Experiment 1, participants performed a conflict task with a virtual co-actor, while the actor-observer relationship was manipulated as a function of the similarity between response modalities. In Experiment 2, the same task was performed both with a virtual and with a human co-actor, while heart-rate measurements were taken to measure the impact of observed conflict on autonomous activity. While both experiments replicated the interpersonal conflict adaptation effects, neither showed evidence of the critical social dimension. We consider the findings as demonstrating that vicarious conflict adaptation does not rely on the social relationship between the actor and co-actor.

## Introduction

Everyday life often requires us to resist temptations that would otherwise distract us from reaching certain goals. Conflict tasks demonstrate the extent to which we find distractions difficult to deal with, or in other words, define how much executive control we need to fulfill task demands. Such tasks include those ones that measure some of the most well-known effects in psychology, for example the Stroop and the Eriksen effects (Stroop, 1935; Eriksen and Eriksen, 1974). Both effects demonstrate the cost of being required to ignore a salient part of the stimulus, i.e., word identities and flanking stimuli. Cognitive control allows us to counter the tempting automaticity, or unravel the confusing interference, so that we may ultimately respond in accordance with the set task goals.

The Simon effect refers to the observation that choice reaction times are delayed if the response location and stimulus location do not correspond (Simon and Rudell, 1967). For example, if a cue such as an arrow pointing left—prompting a left response—is presented on the right side of the screen, it tends to result in a slower, more likely erroneous, response than if it had been presented on the left side. The original explanation of the effect was that it was caused by an automatic tendency to act toward the source of the stimulation (Simon, 1969), although later studies showed that spatial cues do not necessarily attract corresponding responses. In day-to-day life, the consequences of our actions appear where we perform them, causing us to associate actions with corresponding locations. However, after training participants to associate left responses with right-sided action-effects (the appearance of a light on the right side), Hommel (1993) reported inverted Simon effects. Thus, it appears that the appearance of a stimulus primes the intention to act in such a way that has been associated with the action-effect, causing response latency if perception and intention locations do not correspond.

Whatever the source of the conflict, an interesting finding is that conflict effects tend to become smaller after repetition. Thus, across many trials, the higher the proportion of incongruent Stroop stimuli among them, the smaller the effect (Logan and Zbrodoff, 1979), an effect now often referred to as the proportion congruent effect (Lowe and Mitterer, 1982). However, even after a single incongruent Eriksen trial, the subsequent incongruence tends to be resolved faster (Gratton et al., 1992). Likewise with the Simon Effect: if one is to respond left to circles and right to squares, a circle appearing right will constitute a response conflict and, accordingly, slower reactions. The conflict adaption effect (CAE, also called Gratton or sequential compatibility modulation effect) refers to the simple observation that a subsequent square left becomes easier to respond to (e.g., Hommel et al., 2004).

### Social adaptation of conflict control

Interference and inhibition have been considered the mechanisms underlying conflict effects since their first conceptualization (Stroop, 1935, already noted both possibilities), so it is not so strange that such higher order functions are normally invoked by CAE models as well. Broadly speaking, the influential conflict monitoring model (Botvinick et al., 1999, 2001; Botvinick, 2007) as well as models involving inhibitory mechanisms (e.g., Stürmer et al., 2002) rely on dual stimulus-response routes of automaticity and control. A conflict trial such as the previously mentioned circle located right is processed via the automatic, right-location→right-response route. In parallel, a controlled route uses the task instructions to infer the correct, left response from the circular shape. The simultaneously activated response codes are detected, for example in the anterior cingulate cortex (van Veen et al., 2001; Yeung et al., 2004), which prompt the system to engage executive control. This system may then resolve the incompatibility either by facilitating the controlled route or by inhibiting the automatic one. Following successful resolution, one's cognitive state remains prepared for further conflict, and it follows that later conflict effects are smaller.

Although these mechanisms that account for the Simon Effect are normally bound within individuals, a recent modification of the task suggests the effect can also be measured between people. That is, it was noted that if one person is asked to respond solely to the occurrence of a single type of stimulus (e.g., circles), the task effectively becomes a go/no-go task and the Simon Effect disappears. The Social Simon Effect then refers to the observation that if another person (the *co-actor*) is seated at the same desk and asked to share the task by taking care of the other stimulus (e.g., respond right to squares), the Simon Effect reappears (Sebanz et al., 2003). This has been interpreted as a type of shared task-representation, suggesting we effectively “co-represent” the task of the other person, and that perhaps we, on a representational level, do not even separate between the actions of ourselves and those others who share our task (Knoblich and Sebanz, 2006).

If we combine the ideas behind the CAE and the social Simon Effect, the result could be called a social, or *vicarious conflict adaptation effect*. Indeed, it would make strategic sense for one to incorporate observed conflict from another person in order to achieve control over one's own task. Although the effect has not yet been as widely investigated as the CAE or the social Simon Effect, two recent studies indicate that the prediction holds. Winkel et al. (2009) used a modified version of the Simon task and pre-cued left and right located colored circles preceded by a name, to cue the responder in the present trial. Response-feedback was displayed using virtual buttons so that the participant (the actor) could see the actions of someone else (the co-actor). Confirming the hypothesis of vicarious conflict adaptation, they observed reduced conflict effects after merely observing the conflict of the co-actor. They presented collaborating evidence using event related potentials (Winkel et al., 2009) and fMRI (Winkel et al., 2012) to suggest another person's conflict is mentally represented like one's own. In other words, a task co-representation may lead to conflict co-representation, so that to a certain extent one is afflicted by observing a co-actor's conflict and is able to learn from this.

### Feature repetition effects and referential coding

Yet, a significant body of research suggests that neither the control account for the adaptation effect nor the task co-representation account for the Social Simon Effect is without its critics. In terms of the former, lower level feature repetition effects can to a large extent account for conflict adaptation without invoking additional, higher cognitive functions. That is, conflict repetition (incompatible-incompatible, iI, trial sequences) can involve complete stimulus-response repetitions. Stimulus-response repetitions naturally result in priming effects and, consequently, improved performance may be expected (Mayr et al., 2003). On the other hand, both compatible-incompatible (cI) and incompatible-compatible (iC) trial sequences normally involve a partial change of features, which gives rise to delayed responses in a variety of scenarios (Hommel et al., 2001). Together, these observations account for a significant part of the total variance involved in conflict adaptation effects (Hommel et al., 2004). Various strategies for disentangling feature effects from conflict adaptation effects have been proposed, for example by excluding repetitions (Wühr and Ansorge, 2005) or by regression (Notebaert and Verguts, 2007). However, whether such attempts have been successful remains a critical point of debate (Schmidt et al., 2014, 2015).

While the main debate is beyond the scope of the present study, one of its pivotal points lead directly to the present study and will therefore be explained in more detail. That is, if conflict adaptation is the result of a domain general facilitation of attention, then a change in the task, especially if the change is irrelevant, should matter little. However, a change in tasks can remove adaptation effects (Notebaert and Verguts, 2008), and even changes within the same task that are unrelated to conflict can largely eliminate adaptation effects. Thus, Spapé and Hommel (2008) asked participants to respond by saying “high” to high tones and “low” to low tones while listening to task irrelevant voices saying “high” or “low.” Stroop-like effects were found with incongruent tone–word combinations and, unsurprisingly, this conflict effect was smaller after repeating conflict in incompatible-incompatible (iI) sequences. If, however, the voice changed between two trials, for example from a male to a female voice, then this conflict adaptation effect was removed. Spapé and Hommel (2008) interpreted the findings as suggesting that the change in voice disrupted episodic recall, thereby eliminating sequence repetition effects. Other irrelevant changes, such as a rotating display of Simon stimuli (Spapé et al., 2011; Spape and Hommel, 2014) or a change in a self-representing cartoon figure portrayed next to a task (Spapé et al., 2015a) were likewise found to strongly disrupt conflict adaptation effects.

As with pure executive control accounts for conflict adaptation, so too does the higher-level task co-representation account of the Social Simon Effect come with its share of controversy. If, as has been suggested, the Social Simon Effect depends on representing another's task, then the social definition of the task should be important, while changes in the physical aspect of the task should not have any major effects. However, the degree to which the co-actor collaborated in the task had no effect on the Simon Effect, while a large enough co-actor to actor distance eliminated it (Guagnano et al., 2010). Thus, a more parsimonious interpretation of the Social Simon Effect is that the mere presence of salient spatial events can change the reference point in how we spatially represent our own actions (Dolk et al., 2013). This makes the effect “social” to the extent that it implies that the presence of another person is salient enough to affect one's own task, but this is a much weaker stance than the notion of task co-presentation (Dolk et al., 2011).

In sum, the idea that we may represent another person's conflict just like our own and then apply the co-represented conflict to our executive control, is less plausible than it intuitively appears. Even seemingly trivial changes can eliminate conflict adaptation, and a switch between two people is, at least physically, a clear change in a task-relevant feature. The more extreme interpretation of the Social Simon Effect could argue that we are psychologically blind to such changes, since we co-represent the co-actor's task (Sebanz et al., 2003). However, the evidence for task co-representation no longer seems clear-cut, either.

## Experiment 1: effects of action effector on social conflict adaptation

**Figure 1:**
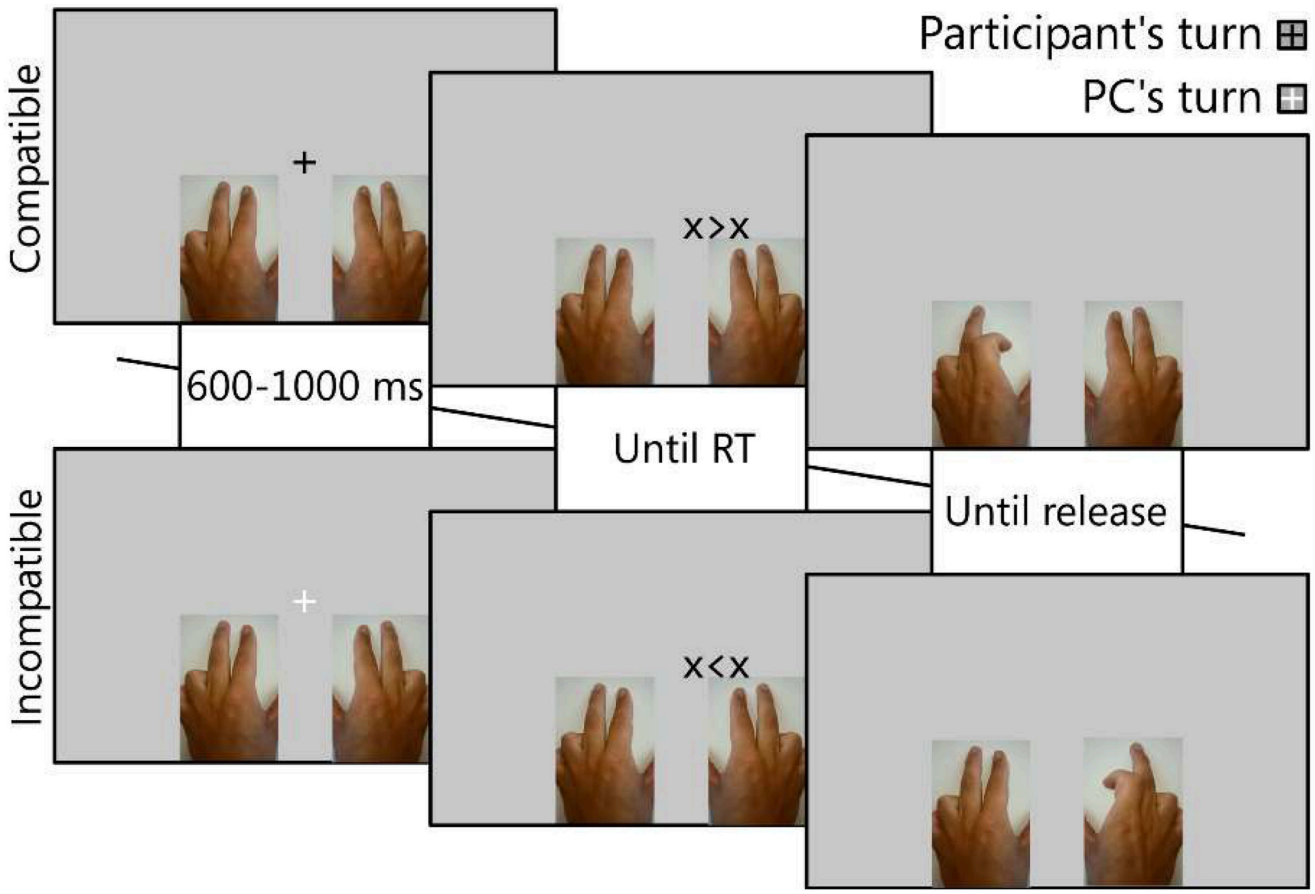
**Trial procedure**. The color of the fixation was used to cue the actor of the present trial. An arrow-like stimulus then appeared, positioned slightly to the left or right of the fixation, pointing toward a **left** (<) or **right** (>) finger response. The hand used by the actor (action effector) was either the same or different: here, the participant (upper panels) used the left hand and the co-actor (PC, lower panels) used the right hand. If the response-location corresponded to the stimulus-location, the trial was compatible (upper panels), otherwise the trial was incompatible (lower panels). Upon the participant's or co-actors reaction, the hand-pictures were adjusted to simulate the pressing of the button.

In order to investigate whether observing a co-actor's conflict is like experiencing our own, we first attempted to replicate the effects found by Winkel et al. (2009, 2012). We maintained most aspects of the task, but slightly modified it to be more consistent with standard Simon tasks. Importantly, we improved trial-to-trial consistency by assigning colors to participants and applying these colors to the fixation cross (see Figure 1) to indicate the turn rather than showing the participant's name. We also enhanced the virtual presence of the co-actor by using photographs of hands, instead of relying on abstract, blinking rectangles.

Additionally, we investigated whether the similarity between the actor and co-actor's action responses affected the vicarious conflict adaptation effect. As described earlier, the association between the response and the anticipated consequences is particularly important for the Simon effect. According to the ideomotor theory of action control, this is because we represent actions by their anticipated consequences (Elsner and Hommel, 2001; Hommel et al., 2001). In theory, then, the degree to which the participant's action-effects correspond to the co-actors action-effects should determine the degree to which task co-representation can occur. If the effect observed by Winkel et al. is based on task co-representation induced conflict adaptation, we would predict that action-effect correspondence should determine the degree to which conflict may “travel” from one person to the next.

Indeed, some recent studies suggested this to be likely. In the Social Transfer of Learning paradigm (Milanese et al., 2010), participants perform a spatial incompatibility task to associate stimulus-locations with the inverse response-locations, before engaging in a Simon task. Interestingly, several social versions of the task have demonstrated that the social and spatial relationship between pairs of participants can have dramatic effects on performance. For example, Iani et al. (2013) asked participants merely to observe computer-generated incompatible responses (i.e., from a virtual co-actor). If, during this time, participants were *potentially* able to act, a type of observational learning was found, transferring to a subsequent Simon task that showed smaller incompatibility effects. Likewise, in a joint version of the task, it was found that the inverse compatibility association carried over to a social Simon Effect, unless participants switched seats before engaging with the Simon task (Ferraro et al., 2012).

It should be underlined that the focus of the present study is not on the Simon effect as such, but on conflict adaptation. Specifically, we are interested in the degree to which observed actions are represented: if action observation is processed to the extent that it can constitute self-experienced conflict, it should modify our own conflict resolution. However, this modulation of conflict adaptation itself should be susceptible to aspects that have been found to affect the social Simon Effect and social transfer of learning.

Thus, we expected that if we represent another's action using our own motor repertoire, then we would expect the degree to which the other's task representation overlaps with one's own to be of critical importance. That is, if two persons have completely different response modalities (e.g., one performs it with their hands, the other with their feet), neither a mirror neuron (Rizzolatti et al., 1996) nor an action co-representation (Sebanz et al., 2003) model would predict simulation of the other's response. Similarly (though perhaps not as strongly as a hand-to-foot transfer), we expected that if the virtual other used a different hand, the vicarious conflict adaptation effect should be reduced.

### Methods

#### Participants

Participants were recruited from the student and members of staff populations at Aalto University, Finland. Six female and 10 male volunteers, age 27.0 ± 3.12 years, participated in the experiment in exchange for one cinema ticket. They were fully informed on the nature and procedure of the experiment and, in accordance with the Declaration of Helsinki, signed informed consent forms prior to their participation. One (male, age 27) participant was found to have less than 50% correct reactions in one condition and was removed from further analysis.

#### Apparatus and stimuli

Experiment design, stimulus display and response collection were conducted using E-Prime 2.0.10.242 professional (Psychology Software Tools, Sharpsburg, PA, USA), running on a Lenovo ThinkPad X1 laptop computer under Microsoft Windows 7 Enterprise x64 SP1. The 14″ display was set to a resolution of 1366 × 768 pixels and a refresh-rate of 60 Hz. Animations of hands were obtained from the first author, who filmed the bending of the index and middle fingers of his left hand and took stills of the extreme points of the movement (see Figure 1) to suggest exaggerated, but clear, button presses. The three pictures (no button pressed, left button pressed, right button pressed) were then horizontally mirrored to create equivalent right-handed pictures and scaled to a size corresponding to a size of 243 × 384 pixels. Response cues consisted of left and right-pointing arrows between two flanking crosses: X < X prompting a left-, and X > X prompting a right finger response. They were positioned 5% (68 pixels) either to the left or right of the center of the screen.

#### Procedure

After receiving written and verbal instructions, the participants completed at least 16 trials to familiarize themselves with the task. Only after they indicated they were confident of their understanding of the task did the experiment commence. At the beginning of each block, the participants were instructed to use either the left (Q and W keys) or the right (O and P keys) hand, though they were asked to keep the other hand on the keyboard as well. As schematically displayed in Figure 1, each trial would begin with a fixation crosshair displayed at the center of the screen for 600–1000 ms (random). The fixation was either red or green, indicating who was responsible for the present trial. Participants were instructed to only respond during this trial if it was “their” color (green or red, counterbalanced). Following, a left or right response cue was displayed to the left- or right- side, until a response was made. Pressing a button would replace the picture of the corresponding hand with one in which the button was shown as pressed. Releasing the button would immediately reset the picture to its default (no button pressed) state. This was shown for a further inter-trial interval (ITI) of 500 ms, unless the participant reacted before the onset of the response cue, 1000 ms after the response cue, or incorrectly, at which point the following feedback was displayed during the ITI: “:(Too early,” “:(Too late,” or “:(Wrong.” The entire experiment took 27.5 ± 3.3 min on average. In conditions in which the participant was instructed to not respond, a virtual response was displayed using the same method as described for the actual participant, with an animation start pre-programmed to commence at the time calculated as the average of the reaction times collected up to that point ± 50 ms, with a button-press duration of 80–150 ms. The virtual participant responded incorrectly at a fixed rate of 1/9 responses to more accurately suggest the behavior of real co-actors.

#### Design

The manual similarity between the actual and virtual participant was varied over 4, randomly ordered, blocks, resulting from the intermixing of the participant's active hand (left or right) and the virtual participant's active hand. Thus, in half the blocks, the action effector of the virtual other was the opposite hand (as in Figure 1) and in the other half, it was the same hand. Each block consisted of 144 trials, designed as 128 trials that were randomized as 64 types of trial pairs across the 4 location (L->L, L->R, R->L, R->R), colors/turns response sequences (2 × 4 × 4 × 4 = 128). Another 16 trials were drawn from a similar sample, with the exception that in these trials, the virtual co-actor was programmed to make an error. The trial-pair paradigm was used as half-way in between the S1/S2 paradigm of feature integration/conflict adaptation designs (c.f. Spapé and Hommel, 2008) and those applying sampling with replacement (Blais, 2008).

The analysis involved a four-way repeated measures analysis of variance (ANOVA) on the reaction times (RTs) with *hand* (same vs. different hand), *previous actor* (same vs. different person), *previous compatibility* (compatible c vs. incompatible i), and *current compatibility* (C vs. I) as factors. However, this analysis can only show whether conflict adaptation changes, not whether there is any remaining. We therefore also tested whether vicarious conflict adaptation was observed by first calculating the conflict adaptation effect (CAE) for each condition using CAE = (cI–cC) – (iI–iC) and then testing these scores against 0. We analyzed the errors in the same way, but as we had no a priori reason to suspect errors should have a different effect, we only report on them if they show divergence from the reaction times, since this could indicate possible speed-accuracy trade-offs.

### Results

Repeated measures ANOVAs testing the hypothesized effect of similarity in action effector between the participant and the virtual participant showed a main effect of *current compatibility* (i.e., Simon effect), *F*_(1, 14)_ = 27.61, *p* < 0.001, $\eta_{p}^{2}$ = 0.66, and a main effect of *previous actor*, *F*_(1, 14)_ = 5.59, *p* < 0.04, $\eta_{p}^{2}$ = 0.29, with incompatible trials being 24 ms slower than compatible trials, and trials following the other actor being 9 ms faster than those following the same actor. The other main effects, of *hand*, *F*_(1, 14)_ = 0.43, *p* > 0.5, $\eta_{p}^{2}$ = 0.03, and *previous compatibility*, *F*_(1, 14)_ = 2.55, *p* > 0.13, $\eta_{p}^{2}$ = 0.15 were both insignificant. A significant interaction between *current compatibility* and *previous compatibility*, *F*_(1, 14)_ = 87.33, *p* < 0.001, $\eta_{p}^{2}=0.86$, was observed, indicative of a CAE. That is, after compatible trials, incompatible trials were ca. 50 ms slower than compatible trials, whereas after incompatible trials, this difference was reduced to 1 ms. Furthermore, a three-way interaction between *previous actor, previous compatibility* and *current compatibility* was found, *F*_(1, 14)_ = 11.85, *p* < 0.004, $\eta_{p}^{2}$ = 0.46. The direction of the effect could be described in a reduction of the CAE if the previous trial was performed by a different actor (see Table 1). However, this effect was not found to be affected by the similarity between observation and execution, as the four-way interaction between *hand, previous actor, previous compatibility* and *current compatibility*, was insignificant, *F*_(1, 14)_ = 0.07, *p* > 0.7, $\eta_{p}^{2}$ < 0.01. No other interaction in the entire factorial design was found significant (*p*s > 0.09, Fs < 3.4).

**Table 1:** **Results Experiment 1**.

**Action effector/hand**	**Previous co-actor**	**c**		**i**		**CAE**
		**cC**	**cI**	**iC**	**iI**	
**REACTION TIME (ms)**						
Same	Same	477 (15)	541 (12)	520 (15)	506 (14)	78 (15)
	Different	472 (15)	513 (15)	496 (14)	503 (15)	33 (11)
Different	Same	475 (15)	535 (12)	517 (15)	504 (17)	73 (15)
	Different	486 (12)	521 (14)	498 (14)	511 (15)	22 (8)
**ERROR (%)**						
Same	Same	0.4 (0.4)	3.1 (1.1)	1.7 (1.4)	0.9 (0.6)	3.5 (1.2)
	Different	0.6 (0.4)	2.1 (0.7)	1.1 (0.6)	0.3 (0.3)	2.3 (1.2)
Different	Same	0.3 (0.3)	2.7 (1.2)	2.9 (1.3)	0.3 (0.3)	4.9 (1.7)
	Different	1.1 (0.8)	1.4 (0.6)	0.7 (0.5)	0.9 (0.6)	0.1 (1.4)

*Provided are means and standard errors of means for each design cell. The conflict adaptation effect (CAE) is computed as a function of the effect of preceding compatibility (c) or incompatibility (i) on current compatibility (C) or incompatibility (I)*.

**Figure 2:**
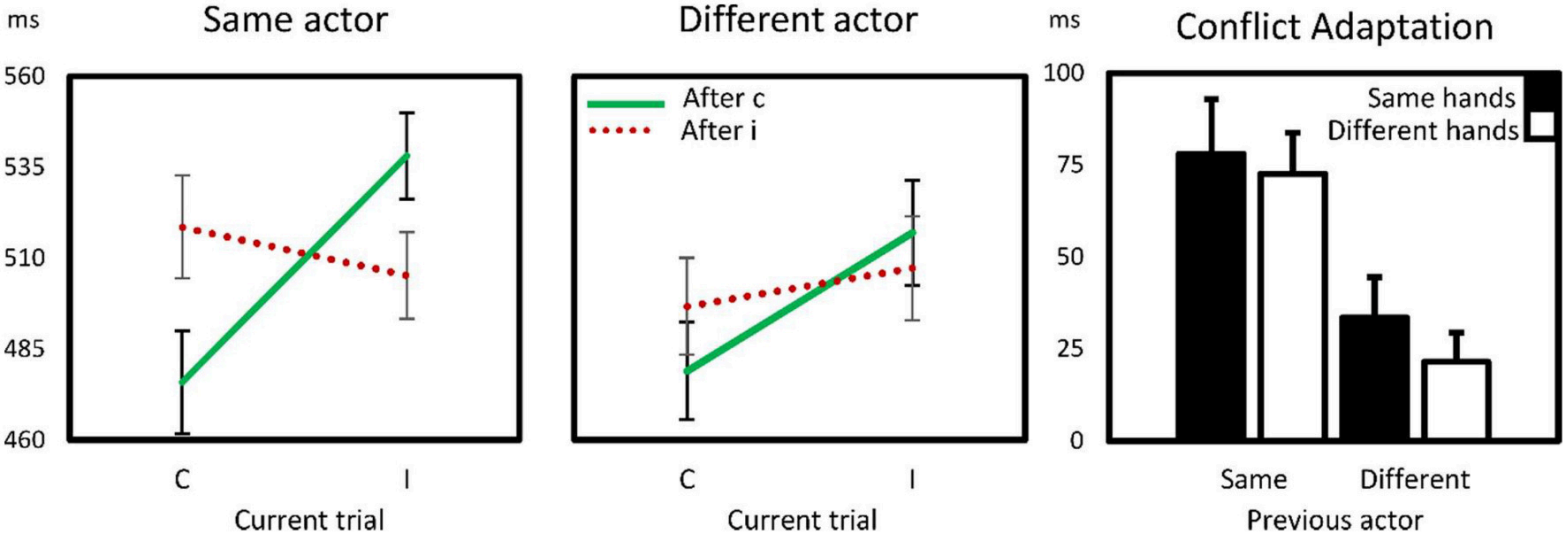
**Sequential compatibility effects within and between actors in Experiment 1**. The first two panels show reaction times for compatible (C) and incompatible (I) trials as a function of preceding trial compatibility. In some trials, the previous actor was the participant him/herself (same actor), in the others, the preceding trial concerned one in which a virtual co-actor responded (different actor). The right panels show the conflict adaptation effects, computed as the interaction effect (cI–cC) – (iI–iC) for same-hand and different-hand trials as a function of previous actor. Error bars are standard errors of the mean CAE.

Thus, as summarized also in Figure 2, a substantial conflict adaptation effect was observed within the same subject. This effect was reduced by up to 72% following trials of the other actor. All CAEs, including those following trials from the other actor were significantly above 0, *t*s (14) > 2.7, *p*s < 0.02, suggesting perhaps a vicarious conflict adaptation exists. However, as *hand* did not enter into any interaction, we found no evidence suggesting that the similarity between action effects had an effect on this vicarious CAE. The CAE with different actors with same hands (the third bar in Figure 2) also did significantly differ from the CAE with different actors with different hands (the fourth bar), *t*_(14)_ = 0.99, *p* > 0.3.

### Conclusion

The experiment replicated the vicarious conflict adaptation effect of a similar magnitude to that observed by Winkel et al. (2012), but two troublesome observations can be made. First, while observing conflict from the virtual other was shown to affect one's own compatibility resolution, it was not quite like undergoing the incompatibility oneself. In fact, after observing, the conflict adaptation effect was only about one third of that after the performing condition. Second, no effect whatsoever was observed regarding the similarity of action effector (same or different hand) between the human and the virtual co-actor. Apparently, the action effector—i.e., the way another “dealt with” the conflict—matters little, suggesting little regard of how the other's conflict is represented.

An inter-individual effect remains, however, but this would be expected even without any social requirement. That is, if we focus purely on the compatibility sequence from an egocentric point of view, one could summarize an observed trial as similar to one in which a stimulus is shown without response requirement. In this way, both this Experiment and the Winkel et al. studies are similar to Hommel et al. (2004)'s Experiment 3. They showed sequential conflict effects occurring even with pairs of trials in which no response was executed on the first trial, just as is the case here. The difference between acting and observing is confounded with the difference between responding and not responding.

In other words, some CAE, even during pure observation, may be expected without social effect. Thus, to answer whether there is any vicarious conflict adaptation, one should go beyond the mere observance of sequential compatibility effects between different persons and provide positive evidence that the social relationship between actor and co-actor modulates the vicarious CAE. Experiment 1 failed to show that the resemblance between actor and co-actor had any effect. Could it be that people simply did not observe the virtual co-actor? This seems unlikely since some vicarious CAE remained. On the other hand, it is possible that the action-effects used here were insufficiently salient in their differences. This seems a reasonable assumption in light of the description of actions: observing the right hand performing an index-fingered response may well have been perceived as a left-sided response.

Of course, a critical difference between Experiment 1 and the studies by Winkel et al. (2012) was that, here, no deception took place: the fact that the virtual actions were computer-generated was entirely clear. Why would someone care about the computer experiencing a conflict? Winkel et al. (2012) clearly found this to be a pertinent question as well, as they took care to make sure participants incorrectly attributed the co-actor's responses to a human being. Furthermore, after completing the experiment, they verified whether participants had seen through the deception (although this was dropped from the analysis). In order, then, to establish whether the effect under investigation is social in nature, it is necessary to show evidence that our conceptualization of who the other is, has a measurable impact.

## Experiment 2: real and virtual others in vicarious conflict adaptation

In Experiment 2, we decided to directly test the effect of the social dimension on the vicarious CAE by recruiting pairs of subjects. Most other aspects of the experiment were the same, but with several improvements resulting from observations made in Experiment 1. We also added physiological recordings to provide further information on conflict processing, particularly with others.

To provide a true test of the social dimension of the effect, we directly tested the “belief” in the other player. To this end, we assigned one fixation color to a participant, one to the computer (as before) and one to another participant. Using dual screen views in separate, neighboring cubicles, we tested the vicarious CAE of each participant, both in self-to-self trials, in virtual co-actor-to-self trials, and in human co-actor-to-self trials, obtaining CAEs for each condition. Clearly, if vicarious conflict adaptation is based on a strong form of action co-representation, one would expect that the type of the other matters (Tsai and Brass, 2007; Tsai et al., 2008). Specifically, there should be a clear difference between virtual co-actor-to-self and other-human-to-self trials, as we expected people to care less about computers than fellow humans (e.g., Ravaja et al., 2006).

Furthermore, we improved the task based on the information gained in Experiment 1. That is, as virtual errors showed little overall effect and as participants did not excessively show inaccurate behavior, we removed the virtual error-trials. We based the virtual actions on the human actions by keeping a standing average of each participant's reaction time (independent of condition). Furthermore, as the action effector had shown very little effect in Experiment 1, we changed the task from a two-finger unimodal version of the Simon task to a more common single-finger bimodal one. This was expected to provide greater salience to the common action-effects between actor and observers. Secondly, this improved the similarity with Winkel et al. (2009)'s design. Thus, the only real difference between virtual and human co-actor scenarios was in the social information regarding the co-actor.

Finally, we additionally acquired heart rate measurements. Changes in autonomic activity, as measured with phasic heart rate measurements, have previously been related to attention regulation (Somsen et al., 2000), error monitoring (Hajcak et al., 2003), and expectation violations (Crone et al., 2003). Thus, in the somewhat rare situation that heart rate is taken into account during a conflict task, Fiehler et al. (2004) found incompatible flanker stimuli to evoke a cardiac deceleration from ca. 1 s after onset. Like Winkel et al. (2009), who collected N2 responses in the ERP, we expected this deceleration to provide information on the degree to which incompatibility evoked an executive response, not only while performing the task oneself, but also while observing another's performance. A strong stance on action co-representation would predict CAEs on heart rate to occur both while performing the task oneself, and while believing someone else to perform the task, but not while knowing the computer performs the task.

### Methods

#### Participants

Thirteen days (*N* = 26, six female, six male, one mixed sex) volunteered to take part in the experiment. Participants were 26.3 ± 2.6 years of age and received a cinema ticket as a compensation for their time.

#### Apparatus and stimuli

The same animations of hands as in Experiment 1 were used, but, given the lack of results related to action effector, we used only the index finger motions of the hands.

#### Procedure

Following instructions, participants were asked to choose a color which would be used to represent themselves during the experiment: red, green or blue. They received 16 training trials to familiarize themselves with the task, after which they completed six experimental blocks of trials. At the beginning of each block, the color cues were used to identify who would participate in the blocks. There were three types of blocks: they would either participate with the other participant (human 1 with human 2 block), participate with the virtual co-actor (human 1 with PC block), or passively observe the other participant with the virtual co-actor (human 2 with PC). Both participants were instructed to use the index fingers of two hands but different keys: one would use the T and I keys for left and right response cues, the other the G and K keys. The trial procedure was otherwise similar to Experiment 1. Trials began with a fixation crosshair that was either in red, green or blue, indicating whose “turn” it was to respond. The entire experiment took 41.3 ± 6.6 min to complete. Full code for one version of the experiment is provided in the Supplementary Information.

#### Physiological data processing

Continuous electrocardiography (ECG) was recorded via bipolar electrodes placed above the manubrium and ninth left rib, and digitized at 1000 Hz using a QuickAmp (BrainProducts GmbH, Gilching, Germany) amplifier. Further pre-processing was done using Matlab (MathWorks, Natick, MA) tools, including filtering the signal at <2 and >100 Hz, with a notch filter at 50 Hz. A local peak detection algorithm was used to detect the R component in the QRS complex of the ECG, which is typically of consistent and high amplitude (here, a threshold of 75% to the median signal was used). Detected peak intervals were then interpolated using spline interpolation with occasionally missing (<2%) intervals excluded. The resulting continuous inter-beat interval (IBI) in milliseconds was then epoched, time-locked to the onset of the critical stimulus onset with 1 s of baseline activity subtracted from the subsequent 4.5 s. The average data was calculated across participant's (active) cC and cI trials (which normally gives the strongest conflict effects) in 100 ms bins. Inspection of the grand average showed an initial cardiac deceleration, likely related to the orienting response (Graham and Clifton, 1966), maximal at 11.2 1100 ms after the stimulus onset for both conditions. Contrasting the two conditions showed a significant difference from 2200–3300 ms [maximally significant at 2900 ms, *t*_(23)_ = 3.11]. As the latency of acceleration and deceleration have previously been shown to provide information on perceptual processing and stimulus significance (Bradley, 2009), we used an analysis with three bins to account for variability in the orienting response, with windowed averages over 0–1100, 1100–2200, and 2200–3300.

#### Design

The type of other was varied between the three block types, with the order randomized and repeating twice. Each block consisted of 128 trials, with 64 types of sequences between two trials, given 4 location, 4 response, and 4 color changes (e.g., red->red, red->green, green->red, green->green).

The analysis of reaction times was similar to Experiment 1, but now with the *type of other* instead of *action effector*. Thus, a single repeated measures ANOVA was used with *type of other* (human co-actor vs. virtual co-actor), *previous actor* (same vs. different person), *previous compatibility* (c vs. i), and *current compatibility* (C vs. I) as factors. Following, we computed CAEs for each combination of *type of other* and *previous actor*, testing them against 0. The hypothesis that vicarious conflict adaptation should be weaker if the other is known to be a virtual co-actor was tested directly with a single paired *T*-test between the different person CAEs for the human vs. virtual co-actor.

The analysis of cardiac changes was similar but included separate five-factor ANOVAs for three distinct scenarios, with for each the factors of *type of other, previous actor, previous compatibility*, and *current compatibility*, as well as *time* (bin 1 vs. bin 2 vs. bin 3). The first scenario was similar to the RT analysis, and concerned the trials in which the participant's own response was required. The second scenario concerned the same blocks, in which the subject participated, but was not presently responding. The third scenario described the situation in which the participant was passively observing the other participant responding. Note that in all three scenarios, the *type of other* is either human or virtual, but with a slight change of meaning: the other is (1) a co-actor (who is not currently acting); (2) a co-actor (who is currently acting); or (3) a non-cooperative actor (who is merely observed).

### Results

#### Behavioral effects

Repeated measures ANOVAs on RTs with *type of other, previous actor, previous compatibility*, and *current compatibility* as factors showed a significant effect of compatibility, *F*_(1, 25)_ = 9.77, *p* = 0.004, $\eta_{p}^{2}$ = 0.28, while *type of other*, *F*_(1, 25)_ = 0.34, *p* > 0.5, $\eta_{p}^{2}$ = 0.01, *previous actor*, *F*_(1, 25)_ = 0.94, *p* > 0.3, and *previous compatibility*, *F*_(1, 25)_ = 0.11, *p* > 0.7, $\eta_{p}^{2}$ < 0.01, did not. Three interactions were found significant. *Previous compatibility* interacted with *current compatibility*, *F*_(1, 25)_ = 86.52, *p* < 0.001, $\eta_{p}^{2}$ = 0.78, reflecting a reduction in the compatibility effect after preceding incompatibility, i.e., a CAE. A significant three-way interaction between *previous actor, previous compatibility* and *current compatibility*, *F*_(1, 25)_ = 32.31, *p* < 0.001, $\eta_{p}^{2}$ = 0.56, indicated that the CAE effect itself was modulated by *previous actor*. *Type of other* interacted only with *previous compatibility*, *F*_(1, 25)_ = 8.67, *p* = 0.007: in PC trials, participants were 2 ms faster after compatible trials; in human trials, they were 8 ms slower. While of interest, this effect was not found to be related to conflict adaptation. That is, neither the interaction between *type of other, previous compatibility*, and *current compatibility*, *F*_(1, 25)_ = 0.42, *p* > 0.5, $\eta_{p}^{2}$ = 0.02, nor the four-way interaction, *F*_(1, 25)_ = 0.42, *p* > 0.9, $\eta_{p}^{2}$ < 0.01, was significant. In other words, we found no evidence in favor of any effect of *type of other* on the CAE.

**Figure 3:**
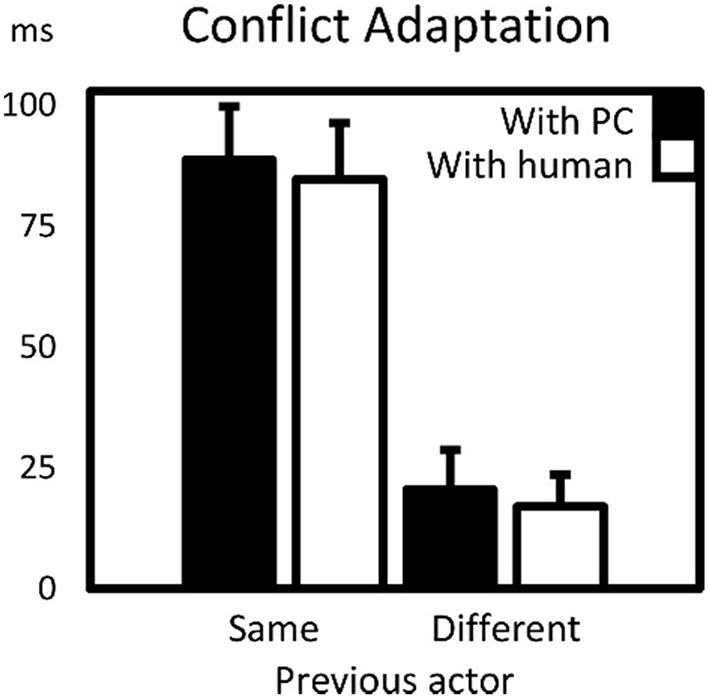
**Conflict adaptation effects (CAEs) for virtual- and human co-actor as a function of previous actor (same or different)**. CAEs were calculated as the interaction term between preceding and current (in)compatibility. Error bars are standard errors of the mean CAE.

As in Experiment 1, all CAEs were larger than 0, *p*s < 0.02, and CAEs with the same actor were much (almost 5x) larger than those with the different actors. However, as indicated by the previously mentioned lack of effect of *type of other* on CAE, the type of different co-actor did not affect CAEs: a simple *post-hoc T*-test did not provide evidence that the CAE with a real co-actor differed from the CAE with the virtual co-actor, *t*_(25)_ = 0.41, *p* > 0.6. CAEs with different actors (the third and fourth bars in Figure 3) were also not significantly different from one another, *t*_(25)_ = 0.36, *p* > 0.7. An overview of the means and standard errors of reaction times and error percentages for every cell in the design is provided in Table 2.

**Table 2:** **Results Experiment 2**.

**Type of co-actor**	**Previous co-actor**	**c**		**i**		**CAE**
		**cC**	**cI**	**iC**	**iI**	
**REACTION TIME (ms)**						
Human	Same	568 (13)	627 (15)	609 (13)	583 (13)	85 (12)
	Different	585 (12)	607 (12)	585 (11)	589 (12)	17 (7)
Virtual	Same	564 (12)	625 (13)	614 (12)	586 (11)	89 (11)
	Different	583 (11)	605 (13)	596 (10)	597 (12)	21 (8)
**ERROR (%)**						
Human	Same	1.7 (0.9)	7.9 (1.7)	3.6 (1.2)	1.4 (0.6)	8.4 (2.2)
	Different	1.7 (0.6)	4.9 (1.4)	1.9 (0.7)	4.4 (1.6)	0.8 (1.4)
Virtual	Same	0.9 (0.5)	2.5 (0.7)	4.6 (1.0)	1.7 (0.8)	4.5 (1.3)
	Different	2.3 (0.9)	3.8 (1.2)	2.7 (0.8)	4.1 (1.1)	0.1 (1.4)

*Provided are means and standard errors of means for each design cell. The conflict adaptation effect (CAE) is computed as a function of the effect of preceding compatibility (c) or incompatibility (i) on current compatibility (C) or incompatibility (I)*.

#### Effects on cardiac response

We first tested whether the effects as observed in the reaction times emerged also in terms of the cardiac response. In a five-way repeated measures ANOVA with *time, type of other, previous actor, previous compatibility*, and *current compatibility* as factors, *time*, *F*_(2, 50)_ = 20.78, *p* < 0.001, $\eta_{p}^{2}$ = 0.45, and *previous actor*, *F*_(1, 25)_ = 11.61, *p* = 0.002, $\eta_{p}^{2}$ = 0.32, were significant. The main factors of *type of other*, *F*_(1, 25)_ = 0.02, *p* > 0.8, $\eta_{p}^{2}$ < 0.01, *previous compatibility*, *F*_(1, 25)_ = 1.20, *p* > 0.2, $\eta_{p}^{2}$ = 0.05, and *current compatibility*, *F*_(1, 25)_ = 0.31, *p* > 0.5, $\eta_{p}^{2}$ = 0.01, however, were not. The effect of *time* could be described as 2 bins with decelerating cardiac activity (of ca. 6.5 ms) followed by acceleration (of ca. 6.6 ms). The effect of *previous actor* could be interpreted as the effect of preparing to respond, which increased deceleration by ca. 8.7 ms. *Previous actor* also interacted with *time*, *F*_(2, 50)_ = 12.76, *p* < 0.001, showing a stronger effect in the first two bins. More interestingly, *time* was found to interact with *type of other*, *F*_(2, 50)_ = 3.67, *p* = 0.03, with deceleration being somewhat stronger in the second bin while being observed by the virtual (7.3 ms) as opposed to human (5.9 ms) other—showing that to some extent, participants were aware of or cared about by whom they were observed.

**Figure 4:**
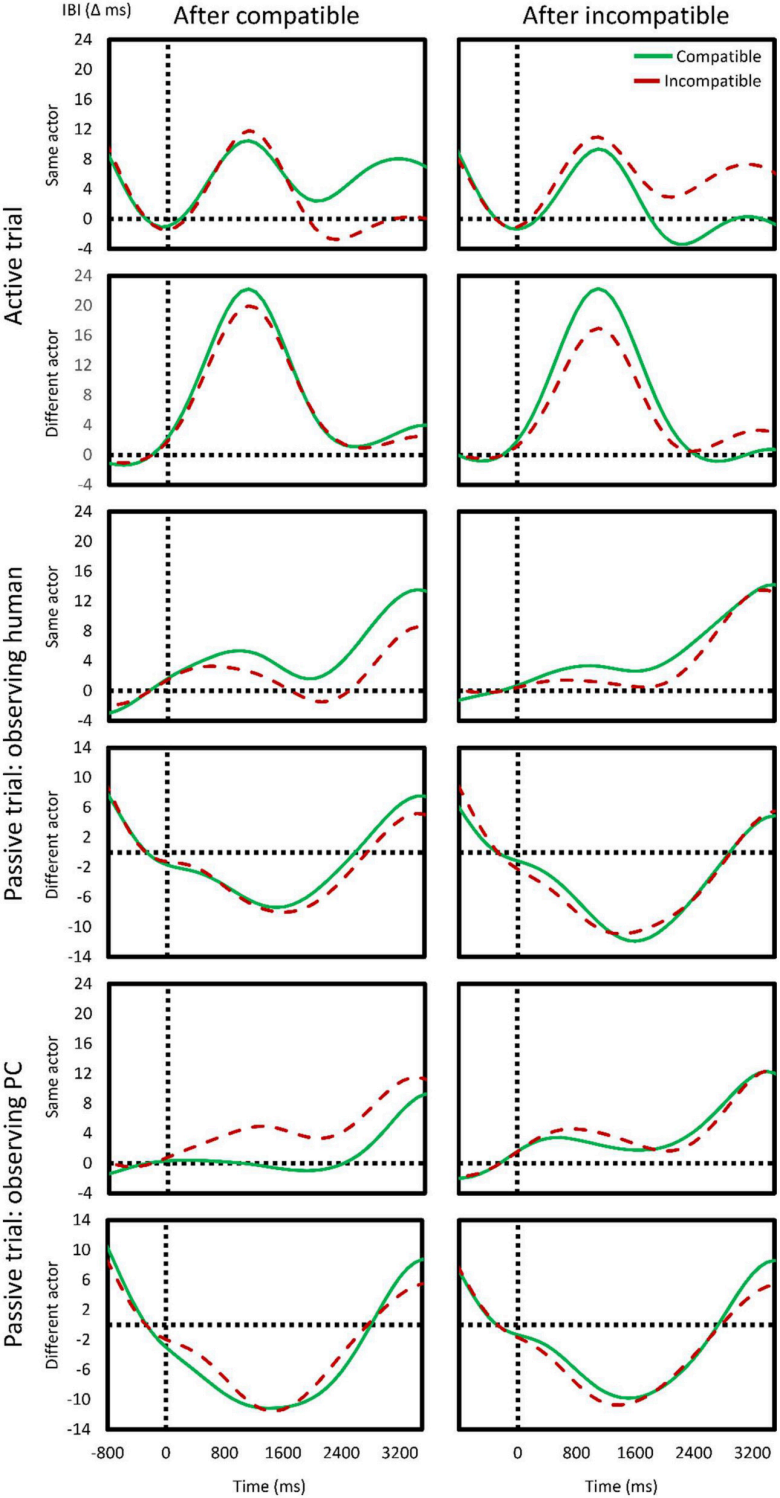
**Effects of compatibility on cardiac response**. The continuous, interpolated change in cardiac inter-beat interval (IBI) is shown for current compatible (green) and incompatible (red) trials as a function of previous compatibility (**left** column: compatible; **right** column: incompatible). The first two rows show the effects in one's own active trial after one's own (first row) or after someone else's (second row) trial. The following four rows show the same, but in the absence of a response (passive trials), and observing as either the real (human) or virtual (PC) other engages in the trial.

However, the critical part of the analysis was in the degree to which this effect related to conflict adaptation. We found a significant interaction between *preceding compatibility* and *current compatibility*, *F*_(1, 25)_ = 4.85, *p* = 0.04, $\eta_{p}^{2}$ = 0.16, indicating a CAE. Furthermore, a significant three-way interaction between *preceding compatibility, current compatibility*, and *time* was observed, *F*_(2, 50)_ = 14.41, *p* < 0.001, $\eta_{p}^{2}$ = 0.37, indicating the CAE changed over time. However, the *type of other* did neither enter a three-way interaction with *previous x current compatibility*, *F*_(1, 25)_ = 0.53, *p* > 0.4, $\eta_{p}^{2}$ = 0.02, nor enter a four-way interaction with *previous* × *current compatibility* × *time*, *F*_(2, 50)_ = 0.17, *p* > 0.8, $\eta_{p}^{2}$ < 0.01. The first two rows of Figure 4 show the direction of the effects described (aggregated over the two *types of others*).

In the second scenario, we tested how the cardiac response was affected while observing the co-acting other's actions. Again, time significantly affected the cardiac activity, *F*_(2, 50)_ = 34.78, *p* < 0.001, $\eta_{p}^{2}$ = 0.58, although here, there was no clear deceleration: instead, acceleration—maximal in the second bin (of 5.1 ms)—was found. *Previous actor* likewise had a significant effect, *F*_(1, 25)_ = 57.80, *p* < 0.001, $\eta_{p}^{2}$ = 0.69, with a change in actor eliciting ca. 8.4 ms of acceleration while a repetition (i.e., of non-participation) resulted in 4.4 ms of deceleration. *Previous compatibility*, *F*_(1, 25)_ = 0.07, *p* > 0.7, $\eta_{p}^{2}$ < 0.01, *current compatibility*, *F*_(1, 25)_ = 0.19, *p* > 0.6, $\eta_{p}^{2}$ = 0.01, and *type of actor*, *F*_(1, 25)_ = 0.90, *p* > 0.3, $\eta_{p}^{2}$ = 0.03, had no significant main effect. No significant interactions were observed. Rows 3–6 in Figure 4 show the effects of a change in actor in this scenario and provide a visual indication of the lack of overall effects of different actors and compatibility.

Finally, in the third scenario, we tested the effects of passively observing the other human co- acting with the virtual other. However, an insignificant main effect of *time*, *F*_(2, 50)_ = 1.08, *p* = 0.31, $\eta_{p}^{2}$ = 0.04, showed that the cardiac response could not reliable be observed. *Current*, *F*_(1, 25)_ = 0.14, *p* > 0.71, $\eta_{p}^{2}$ < 0.01, *and previous compatibility*, *F*_(1, 25)_ = 0.32, *p* > 0.5, $\eta_{p}^{2}$ = 0.01, were insignificant, as was *previous actor*, *F*_(1, 25)_ = 3.47, *p* = 0.07, $\eta_{p}^{2}$ = 0.12. Only one interaction effect in the entire five factor ANOVA was significant: *time* interacted with *preceding compatibility* and *previous actor*, *F*_(2, 50)_ = 4.66, *p* = 0.03, $\eta_{p}^{2}$ = 0.16. This showed that, particularly in the bin 2 and 3, after a compatible trial, a change in actor elicited deceleration whereas a repetition elicited acceleration.

### Conclusion

The results of Experiment 2 are relatively clear-cut: the knowledge that a preceding conflict was being experienced by a real, as opposed to virtual, other had little if any effect. Again, a strong conflict adaptation effect was observed after self-experienced conflict. This effect was strongly reduced if the preceding conflict was not experienced oneself, although not completely eliminated. The question was whether the remainder of the conflict adaptation, which we called the vicarious conflict adaptation effect, was susceptible to the social relationship between actor and co-actor. This was not the case: indeed, if anything, the vicarious conflict adaptation effect was smaller after observing a human-generated trial than after an automatic (virtual) trial.

The effects of experiencing and observing conflict on cardiac response converge with this finding, with little evidence that actively (in scenario 2) or passively (in scenario 3) observing another affected vicarious conflict adaptation. Indeed, while observing, very little effect of conflict on cardiac response could be found: All compatibility-related effects were removed as soon as the trial did not concern the participant him- or herself. This was true whether the participant was observing a human co-actor's trial or a virtual co-actor's trial. The only effect that seemed to be taken into account was whether a trial had a change in actor or not, suggesting that participants did notice the trial-to-trial changes. Even then, the cardiac response was found stronger in trials during which they watched the virtual, as opposed to real, human.

## General discussion

In the present study, we set out to investigate whether a conflict adaptation effect (CAE) can be found not only within individuals, but also between them. Throughout, we found clear conflict (Simon) effects being reduced after incompatible trials. Replicating Winkel and colleagues, we used a social variant of the task, and showed that the CAE can be observed *interindividually*: CAEs were found even after merely observing someone else's conflict. However, as we explained, various different models of the sequential compatibility effects can account for conflict-repetition effects, without necessarily invoking a role for higher cognition such as executive control. The observation of an incompletely reduced CAE therefore cannot in itself be taken as positive evidence for a social dimension. Instead, evidence was sought by investigating whether social factors co-vary with the emergence of the proposed vicarious conflict adaptation effect.

In Experiment 1, we investigated whether correspondence between one's own action-effects and those caused by another would change the vicarious conflict adaptation effect. In general, when we perceive others as similar to us, we are more likely to imitate their behavior (Weatherholtz et al., 2014). According to various theories (e.g., Hurley, 2008), imitation and a range of social cognitive functions are critically dependent on our ability to simulate observed actions using one's own motor repertoire and pre-motor cortex. Thus, we predicted, if the observable effects resulting from someone else's actions map more easily onto those we generate ourselves, then observational learning (somewhat similar to Iani et al., 2013) should be easier. As a result, observed incompatibility should be more like one's own incompatibility, thus increasing the other-to-self (or vicarious) conflict adaptation effect. No effect of effector, however, was found, suggesting little regard for the difference between the action-effects. Whether this means that observed conflict was not vicarious remains hard to say: it is possible that the task co-presentation was not sufficiently evocative (Ferraro et al., 2012), or that the difference between action-effects was not salient enough. On the other hand, if one observes conflict yet does not take into regard how it is dealt with, the added explanatory value of sociality becomes questionable.

Another possibility was that the vicarious conflict adaptation in Experiment 1 never happened: perhaps, the participants' knowledge of the co-actor being an artificial intelligence rather than a real human being reduced the vicarious conflict adaptation by itself. Using two screens and two participants, we changed the experiment by allowing a real other, as well as the previously used virtual other, to co-participate. Previous findings suggest that task co-representation is affected by the social relationship between co-actors (Mussi et al., 2015). Here, however, in terms of vicarious conflict adaptation, no effect concerning the knowledge of the co-actors real or artificial identity was observed. It may therefore be that we observe and co-represent someone else's task without necessarily representing their conflict.

Of course, it is possible that the vicarious conflict adaptation is automatically activated by the action correspondence—i.e., the virtual hands in the present experiment. That is to say, even though participants knew about the virtual other, perhaps they still “cared” about its experience of conflict. Did our subjects perhaps infer some sort of intentionality and a Theory of Mind to the virtual hands, similar to the easy attribution of agency to cartoons of simple line drawings (Abell et al., 2000; Gallagher et al., 2000)? While possible, this explanation seems unlikely given the cardiac IBI data, which only provided a clear effect of the simple observation that a present trial was not one's own trial. At least in terms of autonomic activity, it seems that rather than experiencing the observed conflict of another as one's own, it is instead quickly interpreted as “not my problem.”

It should be noted that our results on the influence of observing someone else's conflict on cardiac response are not in line with previous evidence from functional MRI and EEG. Winkel et al. (2012) showed remarkable overlap between experiencing and observing conflict in frontal areas that have previously been related to executive control. Likewise, Winkel et al. (2009) show observed conflict to result in an event related component that has previously been associated with attentional control (Kopp et al., 1996; Stürmer et al., 2002). It is possible that these cortical correlates are partially or even entirely unrelated to conflict monitoring or resolution—after all, very different psychological functions may involve the same neural structures, an ambiguous situation which gives rise to the logical problem of reverse inference (Poldrack, 2006; Spapé et al., 2015b). On the other hand, it is possible that another's conflict situation is perceived and processed to some extent similar to a self-encountered conflict, but not to the extent that it literally affects the heart as much as it does the brain.

Indeed, one may argue that if we all fully process each other's conflict, we should have difficulty remaining passive. This is a theoretical drawback common to theories that suggest observing others is like personal action. The common coding of self and other (Gallese and Goldman, 1998) may account for a plethora of phenomena, like empathy, imitation (Iacoboni, 2009), language production (Fogassi and Ferrari, 2007), autism (Oberman et al., 2005), and so on. However, the vague boundaries between the personal and interpersonal requires additional mechanisms to account for abilities like self-other discrimination (Uddin et al., 2006) and being able to not imitate (Brass et al., 2009). For our present investigation, it is clear that no matter how their brain processed the stimuli, people who observed another's actions were able to not imitate them and did not seem to be perturbed by their observed conflict. In other words, to complement Winkel et al. (2009): *your conflict matters to me*, but it is not my problem.

## Author contributions

MS: Experiment concept and design, analysis, writing. NR: Experiment concept, writing.

### Conflict of interest statement

The authors declare that the research was conducted in the absence of any commercial or financial relationships that could be construed as a potential conflict of interest. The reviewer DG and handling Editor declared their shared affiliation, and the handling Editor states that the process nevertheless met the standards of a fair and objective review

## References

[B1] Abell F. Happe F. Frith U. (2000). Do triangles play tricks? Attribution of mental states to animated shapes in normal and abnormal development. Cogn. Dev. 15, 1–16. 10.1016/S0885-2014(00)00014-9

[B2] Blais C. (2008). Random without replacement is not random: caveat emptor. Behav. Res. Methods 40, 961–968. 10.3758/BRM.40.4.961 19001387

[B3] Botvinick M. M. Braver T. S. Barch D. M. Carter C. S. Cohen J. D. (2001). Conflict monitoring and cognitive control. Psychol. Rev. 108, 624. 10.1037/0033-295X.108.3.624 11488380

[B4] Botvinick M. M. Nystrom L. E. Fissell K. Carter C. S. Cohen J. D. (1999). Conflict monitoring versus selection-for-action in anterior cingulate cortex. Nature 402, 179–181. 10.1038/46035 10647008

[B5] Botvinick M. M. (2007). Conflict monitoring and decision making: reconciling two perspectives on anterior cingulate function. Cogn. Affect. Behav. Neurosci. 7, 356–366. 10.3758/CABN.7.4.356 18189009

[B6] Bradley M. M. (2009). Natural selective attention: orienting and emotion. Psychophysiology 46, 1–11. 10.1111/j.1469-8986.2008.00702.x 18778317 PMC3645482

[B7] Brass M. Ruby P. Spengler S. (2009). Inhibition of imitative behaviour and social cognition. Philos. Trans. R. Soc. B Biol. Sci. 364, 2359–2367. 10.1098/rstb.2009.0066 19620107 PMC2865080

[B8] Crone E. A. van der Veen F. M. van der Molen M. W. Somsen R. J. van Beek B. Jennings J. R. (2003). Cardiac concomitants of feedback processing. Biol. Psychol. 64, 143–156. 10.1016/S0301-0511(03)00106-6 14602359

[B9] Dolk T. Hommel B. Colzato L. S. Schutz-Bosbach S. Prinz W. Liepelt R. (2011). How ‘Social’ is the social Simon effect? Front. Psychol. 2:84. 10.3389/fpsyg.2011.00084 21687453 PMC3110342

[B10] Dolk T. Hommel B. Prinz W. Liepelt R. (2013). The (not so) social Simon effect: a referential coding account. J. Exp. Psychol. 39, 1248. 10.1037/a0031031 23339346

[B11] Elsner B. Hommel B. (2001). Effect anticipation and action control. J. Exp. Psychol. 27, 229. 10.1037/0096-1523.27.1.229 11248937

[B12] Eriksen B. A. Eriksen C. W. (1974). Effects of noise letters upon the identification of a target letter in a nonsearch task. Percept. Psychophys. 16, 143–149. 10.3758/BF03203267

[B13] Ferraro L. Iani C. Mariani M. Nicoletti R. Gallese V. Rubichi S. (2012). Look what I am doing: does observational learning take place in evocative task-sharing situations? PLoS ONE 7:e43311. 10.1371/journal.pone.0043311 22905256 PMC3419169

[B14] Fiehler K. Ullsperger M. Grigutsch M. von Cramon D. Y. (2004). Cardiac responses to error processing and response conflict, in Errors, Conflicts, and the Brain: Current Opinions on Performance Monitoring, eds Ullsperger M. Falkenstein M. (Leipzig: MPI for Human Cognitive and Brain Sciences), 135–140.

[B15] Fogassi L. Ferrari P. F. (2007). Mirror neurons and the evolution of embodied language. Curr. Dir. Psychol. Sci. 16, 136–141. 10.1111/j.1467-8721.2007.00491.x

[B16] Gallagher H. L. Happé F. Brunswick N. Fletcher P. C. Frith U. Frith C. D. (2000). Reading the mind in cartoons and stories: an fMRI study of ‘theory of mind’ in verbal and nonverbal tasks. Neuropsychologia 38, 11–21. 10.1016/S0028-3932(99)00053-6 10617288

[B17] Gallese V. Goldman A. (1998). Mirror neurons and the simulation theory of mind-reading. Trends Cogn. Sci. (Regul. Ed). 2, 493–501. 10.1016/S1364-6613(98)01262-5 21227300

[B18] Graham F. K. Clifton R. K. (1966). Heart-rate change as a component of the orienting response. Psychol. Bull. 65, 305. 10.1037/h0023258 5325894

[B19] Gratton G. Coles M. G. Donchin E. (1992). Optimizing the use of information: strategic control of activation of responses. J. Exp. Psychol. 121:480. 10.1037/0096-3445.121.4.480 1431740

[B20] Guagnano D. Rusconi E. Umiltà C. A. (2010). Sharing a task or sharing space? On the effect of the confederate in action coding in a detection task. Cognition 114, 348–355. 10.1016/j.cognition.2009.10.008 19914615

[B21] Hajcak G. McDonald N. Simons R. F. (2003). To err is autonomic: error-related brain potentials, ANS activity, and post-error compensatory behavior. Psychophysiology 40, 895–903. 10.1111/1469-8986.00107 14986842

[B22] Hommel B. Müsseler J. Aschersleben G. Prinz W. (2001). The Theory of Event Coding (TEC): a framework for perception and action planning. Behav. Brain Sci. 24, 849–878; discussion 878–937. 10.1017/S0140525X01000103 12239891

[B23] Hommel B. Proctor R. W. Vu K.-P. L. (2004). A feature-integration account of sequential effects in the Simon task. Psychol. Res. 68, 1–17. 10.1007/s00426-003-0132-y 14752663

[B24] Hommel B. (1993). Inverting the Simon effect by intention. Psychol. Res. 55, 270–279. 10.1007/BF00419687 8416040

[B25] Hurley S. (2008). The shared circuits model (SCM): How control, mirroring, and simulation can enable imitation, deliberation, and mindreading. Behav. Brain Sci. 31, 1–22. 10.1017/S0140525X07003123 18394222

[B26] Iacoboni M. (2009). Imitation, empathy, and mirror neurons. Annu. Rev. Psychol. 60, 653–670. 10.1146/annurev.psych.60.110707.163604 18793090

[B27] Iani C. Rubichi S. Ferraro L. Nicoletti R. Gallese V. (2013). Observational learning without a model is influenced by the observer's possibility to act: evidence from the Simon task. Cognition 128, 26–34. 10.1016/j.cognition.2013.03.004 23583542

[B28] Knoblich G. Sebanz N. (2006). The social nature of perception and action. Curr. Dir. Psychol. Sci. 15, 99–104. 10.1111/j.0963-7214.2006.00415.x

[B29] Kopp B. Rist F. Mattler U. W. E. (1996). N200 in the flanker task as a neurobehavioral tool for investigating executive control. Psychophysiology 33, 282–294. 10.1111/j.1469-8986.1996.tb00425.x 8936397

[B30] Logan G. D. Zbrodoff N. J. (1979). When it helps to be misled: facilitative effects of increasing the frequency of conflicting stimuli in a Stroop-like task. Mem. Cogn. 7, 166–174. 10.3758/BF03197535

[B31] Lowe D. G. Mitterer J. O. (1982). Selective and divided attention in a Stroop task. Can. J. Psychol. 36, 684. 10.1037/h0080661 7159848

[B32] Mayr U. Awh E. Laurey P. (2003). Conflict adaptation effects in the absence of executive control. Nat. Neurosci. 6, 450–452. 10.1038/nn1051 12704394

[B33] Milanese N. Iani C. Rubichi S. (2010). Shared learning shapes human performance: transfer effects in task sharing. Cognition 116, 15–22. 10.1016/j.cognition.2010.03.010 20381024

[B34] Mussi D. R. Marino B. F. Riggio L. (2015). The influence of social and nonsocial variables on the Simon effect. Exp. Psychol. 62, 215–231. 10.1027/1618-3169/a000292 26421448

[B35] Notebaert W. Verguts T. (2007). Dissociating conflict adaptation from feature integration: a multiple regression approach. J. Exp. Psychol. 33:1256. 10.1037/0096-1523.33.5.1256 17924821

[B36] Notebaert W. Verguts T. (2008). Cognitive control acts locally. Cognition 106, 1071–1080. 10.1016/j.cognition.2007.04.011 17537419

[B37] Oberman L. M. Hubbard E. M. McCleery J. P. Altschuler E. L. Ramachandran V. S. Pineda J. A. (2005). EEG evidence for mirror neuron dysfunction in autism spectrum disorders. Cogn. Brain Res. 24, 190–198. 10.1016/j.cogbrainres.2005.01.014 15993757

[B38] Poldrack R. A. (2006). Can cognitive processes be inferred from neuroimaging data? Trends Cogn. Sci. (Regul. Ed). 10, 59–63. 10.1016/j.tics.2005.12.004 16406760

[B39] Ravaja N. Saari T. Turpeinen M. Laarni J. Salminen M. Kivikangas M. (2006). Spatial presence and emotions during video game playing: does it matter with whom you play? Presence Teleoper. Virtual. Environ. 15, 381–392. 10.1162/pres.15.4.381

[B40] Rizzolatti G. Fadiga L. Gallese V. Fogassi L. (1996). Premotor cortex and the recognition of motor actions. Cogn. Brain Res. 3, 131–141. 10.1016/0926-6410(95)00038-0 8713554

[B41] Schmidt J. R. De Schryver M. Weissman D. H. (2014). Removing the influence of feature repetitions on the congruency sequence effect: why regressing out confounds from a nested design will often fall short. J. Exp. Psychol. 40, 2392. 10.1037/a0038073 25419672

[B42] Schmidt J. R. Notebaert W. Van Den Bussche E. (2015). Is conflict adaptation an illusion? Front. Psychol. 6:72. 10.3389/fpsyg.2015.00172 25762962 PMC4332165

[B43] Sebanz N. Knoblich G. Prinz W. (2003). Representing others' actions: just like one's own? Cognition 88, B11–B21. 10.1016/S0010-0277(03)00043-X 12804818

[B44] Simon J. R. Rudell A. P. (1967). Auditory SR compatibility: the effect of an irrelevant cue on information processing. J. Appl. Psychol. 51, 300. 10.1037/h0020586 6045637

[B45] Simon J. R. (1969). Reactions toward the source of stimulation. J. Exp. Psychol. 81, 174. 10.1037/h0027448 5812172

[B46] Somsen R. J. Van der Molen M. W. Jennings J. R. van Beek B. (2000). Wisconsin card sorting in adolescents: analysis of performance, response times and heart rate. Acta Psychol. (Amst). 104, 227–257. 10.1016/S0001-6918(00)00030-5 10900707

[B47] Spapé M. M. Hommel B. (2008). He said, she said: episodic retrieval induces conflict adaptation in an auditory Stroop task. Psychon. Bull. Rev. 15, 1117–1121. 10.3758/PBR.15.6.1117 19001577

[B48] Spape M. M. Hommel B. (2014). Sequential modulations of the Simon effect depend on episodic retrieval. Front. Psychol. 5:855. 10.3389/fpsyg.2014.00855 25152743 PMC4126466

[B49] Spapé M. M. Ahmed I. Jacucci G. Ravaja N. (2015a). The self in conflict: actors and agency in the mediated sequential Simon Task. Front. Psychol. 6:304. 10.3389/fpsyg.2015.00304 25852618 PMC4370107

[B50] Spapé M. M. Band G. P. H. Hommel B. (2011). Compatibility-sequence effects in the Simon task reflect episodic retrieval but not conflict adaptation: evidence from LRP and N2. Biol. Psychol. 88, 116–123. 10.1016/j.biopsycho.2011.07.001 21767598

[B51] Spapé M. M. Filetti M. Eugster M. J. Jacucci G. Ravaja N. (2015b). Human computer interaction meets psychophysiology: a critical perspective, in Symbiotic Interaction, eds Blankertz B. Jacucci G. Gamberini L. Spagnolli A. Freeman J. (Cham: Springer), 145–158.

[B52] Stroop J. R. (1935). Studies of interference in serial verbal reactions. J. Exp. Psychol. 18, 643 10.1037/h0054651

[B53] Stürmer B. Leuthold H. Soetens E. Schröter H. Sommer W. (2002). Control over location-based response activation in the Simon task: behavioral and electrophysiological evidence. J. Exp. Psychol. 28:1345. 10.1037/0096-1523.28.6.1345 12542132

[B54] Tsai C.-C. Brass M. (2007). Does the human motor system simulate Pinocchio's actions? Coacting with a human hand versus a wooden hand in a dyadic interaction. Psychol. Sci. 18, 1058–1062. 10.1111/j.1467-9280.2007.02025.x 18031412

[B55] Tsai C.-C. Kuo W.-J. Hung D. L. Tzeng O. J. (2008). Action co-representation is tuned to other humans. J. Cogn. Neurosci. 20, 2015–2024. 10.1162/jocn.2008.20144 18416679

[B56] Uddin L. Q. Molnar-Szakacs I. Zaidel E. Iacoboni M. (2006). rTMS to the right inferior parietal lobule disrupts self–other discrimination. Soc. Cogn. Affect. Neurosci. 1, 65–71. 10.1093/scan/nsl003 17387382 PMC1832105

[B57] van Veen V. Cohen J. D. Botvinick M. M. Stenger V. A. Carter C. S. (2001). Anterior cingulate cortex, conflict monitoring, and levels of processing. Neuroimage 14, 1302–1308. 10.1006/nimg.2001.0923 11707086

[B58] Weatherholtz K. Campbell-Kibler K. Jaeger T. F. (2014). Socially-mediated syntactic alignment. Lang. Var. Change 26, 387–420. 10.1017/S0954394514000155

[B59] Winkel J. Wijnen J. G. Danielmeier C. Groen I. I. Derrfuss J. Ridderinkhof K. R.. (2012). Observed and self-experienced conflict induce similar behavioral and neural adaptation. Soc. Neurosci. 7, 385–397. 10.1080/17470919.2011.628760 22017337

[B60] Winkel J. Wijnen J. G. Ridderinkhof K. R. Groen I. I. Derrfuss J. Danielmeier C.. (2009). Your conflict matters to me! Behavioral and neural manifestations of control adjustment after self-experienced and observed decision-conflict. Front. Hum Neurosci. 3:57. 10.3389/neuro.09.057.2009 20198103 PMC2802321

[B61] Wühr P. Ansorge U. (2005). Exploring trial-by-trial modulations of the Simon effect. Q. J. Exp. Psychol. 58, 705–731. 10.1080/02724980443000269 16104103

[B62] Yeung N. Botvinick M. M. Cohen J. D. (2004). The neural basis of error detection: conflict monitoring and the error-related negativity. Psychol. Rev. 111:931. 10.1037/0033-295X.111.4.931 15482068

