# Supplementary figures and images for: Not My Problem: Vicarious Conflict Adaptation with Human and Virtual Co-actors
Source: Front Psychol. 2016 Apr 28;7:606. doi: 10.3389/fpsyg.2016.00606 (PMC4848756; doi:10.3389/fpsyg.2016.00606)

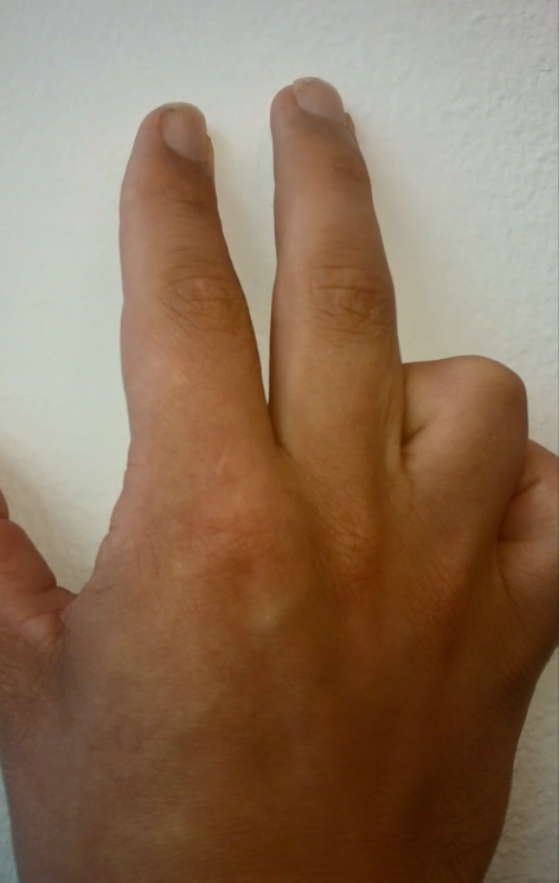

Supplement: Supplementary file 1 [file Experimentsourcecode(E-Prime).zip › RIGHT_ND2.bmp]

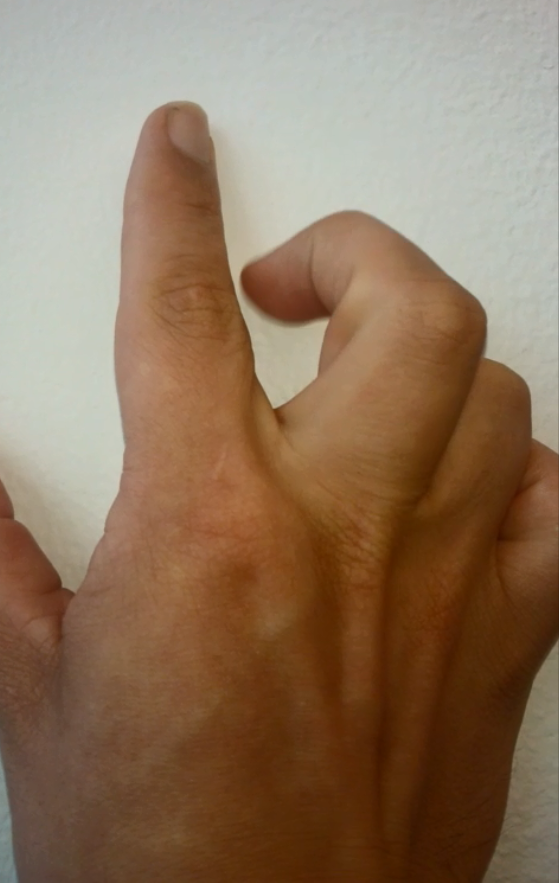

Supplement: Supplementary file 1 [file Experimentsourcecode(E-Prime).zip › RIGHT_RD.bmp]

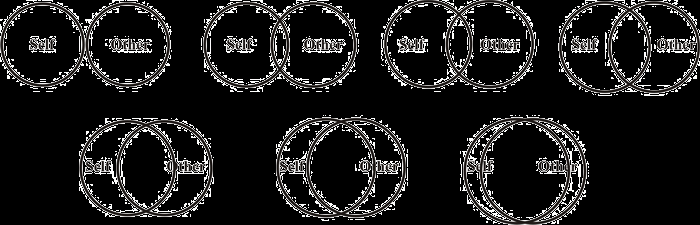

Supplement: Supplementary file 1 [file Experimentsourcecode(E-Prime).zip › IOS.bmp]

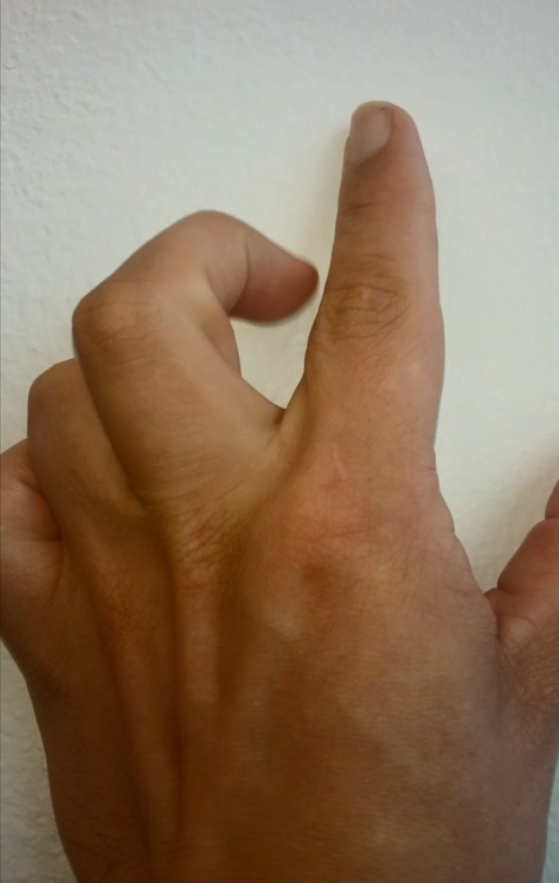

Supplement: Supplementary file 1 [file Experimentsourcecode(E-Prime).zip › LEFT_LD.bmp]

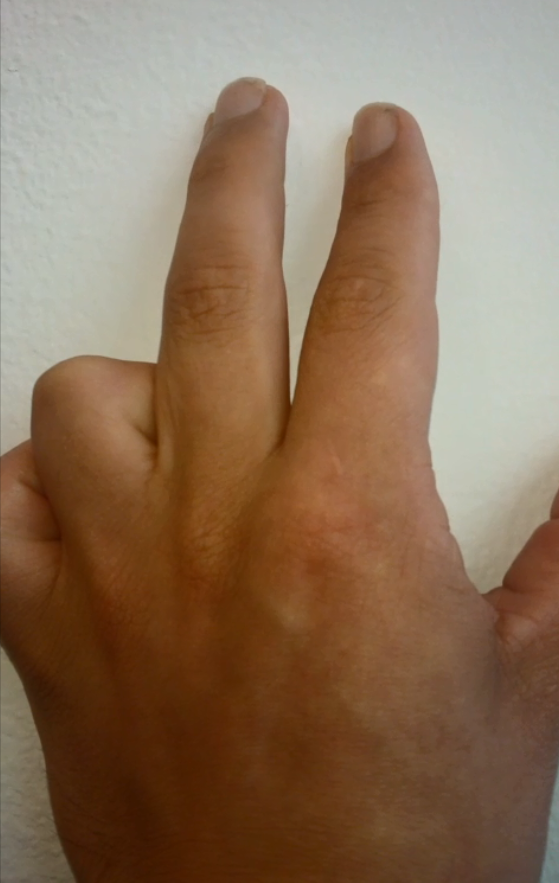

Supplement: Supplementary file 1 [file Experimentsourcecode(E-Prime).zip › LEFT_ND.bmp]

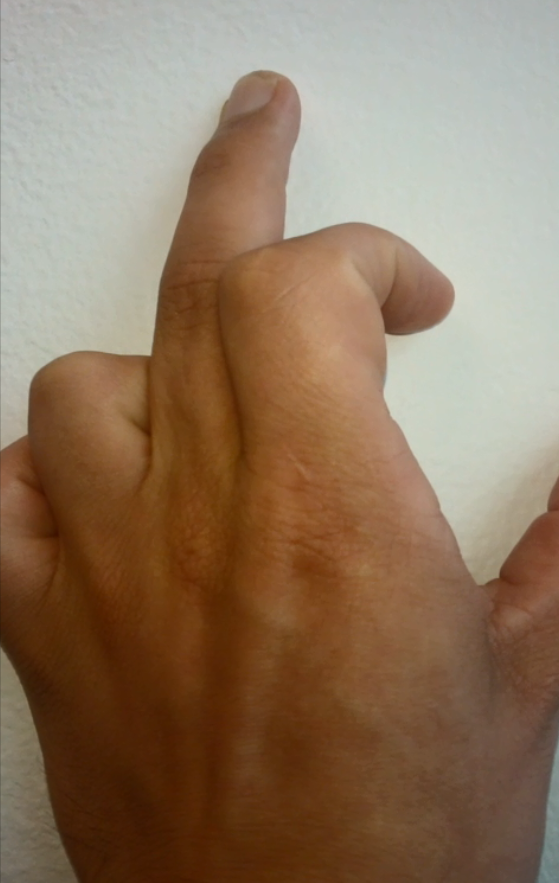

Supplement: Supplementary file 1 [file Experimentsourcecode(E-Prime).zip › LEFT_RD.bmp]

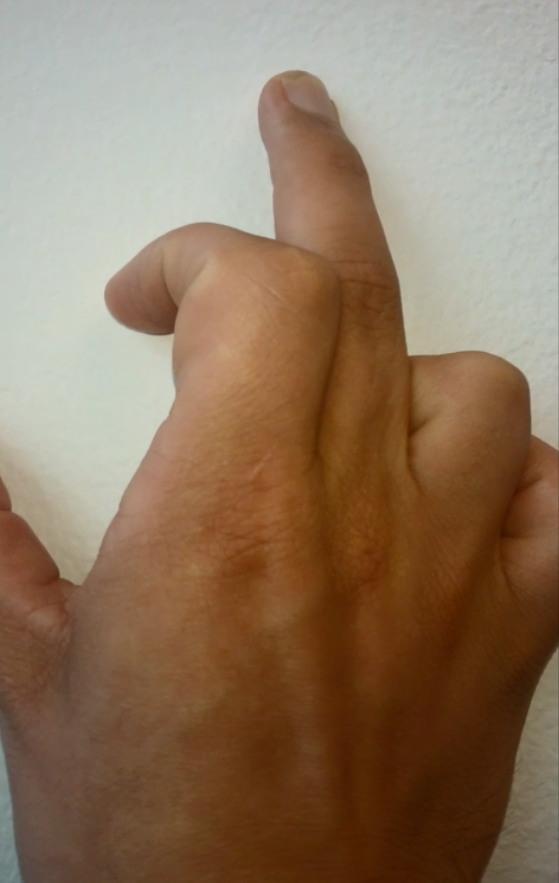

Supplement: Supplementary file 1 [file Experimentsourcecode(E-Prime).zip › RIGHT_LD.bmp]
